# Quality of Life in Papillary Thyroid Microcarcinoma Patients Undergoing Radiofrequency Ablation or Surgery: A Comparative Study

**DOI:** 10.3389/fendo.2020.00249

**Published:** 2020-05-15

**Authors:** Yu Lan, Yukun Luo, Mingbo Zhang, Zhuang Jin, Jing Xiao, Lin Yan, Yaqiong Zhu

**Affiliations:** ^1^School of Medicine, Nankai University, Tianjin, China; ^2^Department of Ultrasound, The First Medical Center of Chinese PLA General Hospital, Beijing, China; ^3^Department of Ultrasound, The People's Hospital of Liaoning Province, Shenyang, China

**Keywords:** papillary thyroid microcarcinoma (PTMC), ultrasound, radiofrequency ablation (RFA), surgery, quality of life

## Abstract

**Objective:** Papillary thyroid microcarcinoma (PTMC) has a good prognosis and a long survival time. Health-related quality of life (HRQoL) is vital for PTMC patients during their survivorship. Ultrasound (US)-guided radiofrequency ablation (RFA), which has high efficacy and safety, is recommended as an alternative treatment to surgery for the patients with low-risk PTMC. However, the assessment of QoL of patients with PTMC has not been specially reported. The purpose of our study was to compare the HRQoL of patients with PTMC who underwent RFA and those who underwent surgery.

**Methods:** From October 2019 to December 2019, 88 PTMC patients were enrolled in our study, including 54 in RFA group and 34 in surgery group. We used three questionnaires which included the 36-item short form health survey (SF-36), thyroid cancer-specific quality of life (THYCA-QOL), and Fear of Progression Questionnaire-Short Form (FoP-Q-SF) for each patient to evaluate their scores of HRQoL. The scores were compared after adjusting for age, sex, medical expense, and follow-up time.

**Results:** According to the SF-36, the scores of the domain for the role limitation due to physical problems and emotional problems (RP, RE) as well as Physical Component Summary (PCS) showed a significant negative linear association between the RFA group and surgery group: RP coefficient [coef]−22.613 [confidence interval (CI) −33.504 to −11.723], *p* < 0.001, RE (coef: −21.901 [CI −36.737 to −7.064], *p* = 0.004), and PCS (coef: −8.312 [CI −13.694 to −2.930], *p* = 0.003). The THYCA-QOL showed that the scores of the surgery group were higher than that of the RFA group regarding scars (coef: 10.246 [CI 1.330 to 19.162], *p* = 0.025 according to the multivariate analysis), suggesting a higher level of complaint in the surgery group. There was no statistically significant difference in the scores of FoP-Q-SF between the two groups.

**Conclusions:** In patients with PTMC, US-guided RFA offers advantage over surgery in terms of HRQoL, which supports the role of RFA as an alternative strategy to surgery.

## Introduction

Papillary thyroid microcarcinoma (PTMC), which is papillary thyroid carcinoma (PTC) with a maximum diameter of ≤ 1 cm (1, 2), has high incidence, low mortality rate, and over 90% 10-year survival rate (3–6). Although it is often referred to as the “good cancer,” health-related quality of life (HRQoL) is often reduced by sleep disorders, fatigue, and limited daily activity compared with general people (7, 8). Studies showed that HRQoL of thyroid cancer survivors is negatively affected for up to 20 years after treatment (9). Thus, more attention should be paid to the HRQoL of patients when choosing treatment strategy (10).

The recent American Thyroid Association (ATA) Guidelines also emphasize that physicians should consider long-term quality of life outcomes when making treatment decisions, and recommend active surveillance (AS) for PTMC (2). However, patients who worry about untreated tumors or tumor metastases may experience significant psychological stress (11). Although surgery is a general recommendation of treatment (2, 12–14), it may cause some complications such as permanent recurrent laryngeal nerve paralysis, hypothyroidism, hypoparathyroidism, or an ugly scar (15–19), which may decrease QoL of thyroid cancer survivors and negatively affect their psychological well-being and social function (7, 20).

Ultrasound (US)-guided thermal ablation has become a new treatment strategy for thyroid nodules (21–25). The efficacy and safety of microwave ablation and radiofrequency ablation (RFA) in the treatment of thyroid microcarcinoma have been demonstrated in previous studies (26–28). However, the comparative study of the quality of life for thyroid cancer patients between undergoing surgery and thermal ablation has not been reported yet.

In this study, we sought to make the comparison of HRQoL between the low-risk PTMC patients who underwent US-guided RFA and surgery by using three different and validated questionnaires.

## Materials and Methods

### Patients

The comparative study was approved by the Institutional Review Board at General Hospital of Chinese PLA (S2019-211-01). Informed consent for treatment and questionnaire procedures was obtained from each participant. From October 2019 to December 2019, 88 patients (15 male and 73 female with an average age of 42.09 years ± 10.02 years ranging from 23 years to 64 years) who had undergone RFA or open surgery in our hospital were enrolled in our study (Figure 1A).

**Figure 1A:**
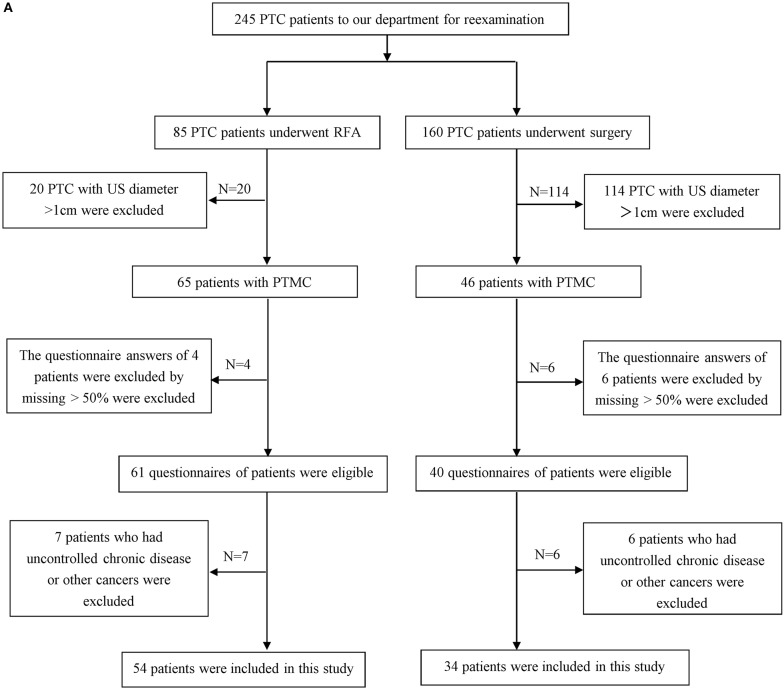
The flow chart of inclusion and exclusion of the RFA and surgery group. PTC, papillary thyroid carcinoma; PTMC, papillary thyroid microcarcinoma; US, ultrasound.

For the RFA group, patients were enrolled in this study if they fulfilled the following criteria: (1) a solitary suspicious thyroid nodule with a maximum diameter of less than 1 cm was detected by US; (2) low-risk PTMC, which is defined as patients without any evidence of nodal or distant metastases, extrathyroidal extension, or history of radiation exposure (2, 12); (3) core needle biopsy (CNB) confirming PTMC without aggressive histological type (except hobnail, poorly differentiated, or tall cell variants) (29); (4) patients refused surgery or were poor candidates for surgery; and (5) more than 1 month's follow-up.

For the surgery group, patients had undergone surgery and were included in this study if they fulfilled the following criteria: (1) a solitary suspicious thyroid nodule with a maximum diameter of ≤ 1 cm detected by pre-operative US; (2) low-risk PTMC, which is defined as patients without any evidence of nodal or distant metastases, extrathyroidal extension, or history of radiation exposure (2, 12); (3) surgical pathology confirming PTMC without aggressive histological type and no other aggressive features; and (4) more than 1 month's follow-up.

A total of 54 PTMC patients underwent RFA, two of whom were poor candidates for surgery due to chronic cardiac insufficiency, and 52 of whom subjectively refused open surgery or active surveillance. 34 PTMC patients underwent surgery, with 16 patients who underwent total thyroidectomy and 18 patients who underwent unilateral lobectomy.

All PTC patients who underwent surgery or RFA came to our department for review and filled in the questionnaire, and the questionnaire screening process was completed by the investigator (Y.L.) alone. The characteristics of tumors and surgical methods of the patients were obtained from the electronic medical record system of our hospital, and the questionnaires of the patients meeting the inclusion criteria were enrolled in this study (Figure 1B).

**Figure 1B:**
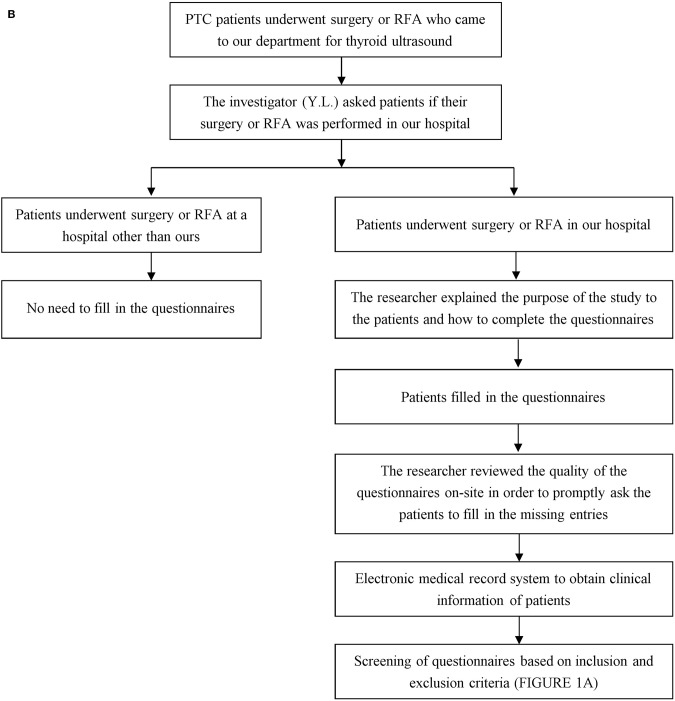
The flow chart of follow-up process.

### Ablation Procedure

In our study, all patients underwent routine US and contrast-enhanced ultrasonography (CEUS) examination before ablation by using a 15L8W linear array transducer and a real-time US System (Siemens ACUSON Sequoia 512, Siemens, Mountain View, CA), a L12-5 linear array transducer and a real-time US System (Philips EPIQ7, Philips Healthcare, Bothell, WA), or a L12-4 linear array transducer and a real-time US System (Mindray M9, Mindray, Shenzhen, China). Routine US characteristics of lesions including location, composition, echogenicity, shape, margin, and echogenic foci (Table 1) were assessed and recorded according to the ACR TI-RADS (30). All RFA procedures were performed by the same sonographer (K.Y.L.), who has 20 years' clinical experience in routine and interventional US.

**Table 1:** Tumor characteristics of RFA and surgery group.

**Characteristics**	**Ablation group (*n* = 54)**	**Surgery group (*n* = 34)**	***p*-value**
Location			0.380
Left	32	15	
Right	21	18	
Isthmus	1	1	
Composition			/
Cystic or almost completely cystic	0	0	
Spongiform	0	0	
Mixed cystic and solid	0	0	
Solid or almost completely solid	54	34	
Echogenicity			0.154
Anechoic	0	0	
Hyperechoic or isoechoic	0	0	
Hypoechoic	41	30	
Very hypoechoic	13	4	
Shape			
Wider-than-tall	19	8	
Taller-than-wide	35	26	
Margin			0.094
Smooth	20	6	
Ill-defined	11	6	
Lobulated or irregular	23	22	
Extra-thyroidal extension	0	0	
Echogenic foci			0.231
None or large comet-tail artifacts	33	15	
Macrocalcifications	0	0	
Peripheral(rim)calcifications	1	2	
Punctate echogenic foci	20	17	

Patients lay in the supine position with their necks extended. Skin sterilization was performed and 1% lidocaine was used for local anesthesia at the intended puncture site. The hydrodissection technique was used with a mixture of 1% lidocaine injected into the anterior capsule space and normal saline injected into the posterior capsule space to protect vital structures (cervical artery, trachea, esophagus, recurrent laryngeal nerve) to prevent thermal injury when the distance between the lesion and the surrounding vital structures was <5 mm. Moving-shot ablation technique was used to perform the RFA. (22, 25). The RFA extends beyond the edge of the lesion to prevent local residue and recurrence. When all the target areas become transient hyperechoic zones, the ablation stops. During the procedure, more attention would be paid to protecting the surrounding vital structures to prevent serious complications such as hematoma formation or nerve injury. After RFA, all patients were observed for 1 to 2 h and were evaluated for any complications during or immediately after RFA operation.

### Surgery Procedure

Patients in the surgery group were operated on by surgeons (W.T. or Z.Q.) with more than 20 years of experience in thyroid surgery. They complete more than 1,000 surgical procedures each year. Surgical procedures are performed according to the ATA guidelines (2), including unilateral lobectomy or total thyroidectomy with or without cervical lymph node dissection and iodine therapy.

### Demographic and Clinical Characteristics

Demographic characteristics, such as age, sex, height, weight, marital status, education level, employment status, source of medical expenses, and place to live, were collected. Clinical characteristics such as data regarding levothyroxine (LT4) supplementation, comorbidity, and family history of thyroid cancer were collected.

### HRQoL Questionnaires

#### Short-Form Survey

The SF-36 (Chinese version) is a multi-purpose short form survey, a well-validated and standardized questionnaire measuring HRQoL that is used in many publications (31–35). It consists of 36 questions measuring eight domains: physical functioning (PF), role-physical (RP), bodily pain (BP), general health (GH), vitality (VT), social functioning (SF), role-emotional (RE), and mental health (MH). Two total scores can be calculated: physical component summary (PCS) and mental component summary (MCS) representing the physical wellbeing and emotional wellbeing, respectively. All scores of domains are transformed to scales of 0 to 100. The higher scores on the domains indicate lower disability and better HRQoL.

#### Thyroid Cancer-Specific Quality of Life Questionnaire (THYCA-QOL)

The Chinese version of THYCA-QOL was used to assess the thyroid-specific symptoms resulting from the thyroid cancer itself or its treatment (36). It includes 24 questions measuring seven symptom domains (neuromuscular, voice, attention, sympathetic, throat/mouth, psychological, and sensory symptoms), as well as six single scales (scar, feeling cold, tingling sensation, weight gain, headache, and reduced sexual interest) (7, 37). The questionnaire has a strict time scale (4 weeks for the sexual interest item, 1 week for the other items). All items are classified into four levels (1 = “not at all,” 2 = “a little,” 3 = “quite a bit,” and 4 = “very much”) and are counted as 1 to 4 points. A higher score represents more complaints and worse HRQoL caused by that symptom (37).

#### Fear of Progression Questionnaire

This questionnaire was developed by Mehnert et al. (38) and has been applied to patients with systemic sclerosis (39) and cancer (40) with high reliability and validity. It consists of 12 items measuring two domains (physiological health dimension and social family dimension). The Likert 1 to 5 score method is adopted. Each item is scored from 1 to 5: “never” to “often.” The scale is self-rated by patients with a total score of 12 to 60 points. A higher score indicates a greater level of anxiety about disease progression.

All questionnaires in this study were sent and received by the investigator (Y.L.), who explained the method of filling in the questionnaires. The three questionnaires mentioned above were completed after obtaining the patients' informed consent. The researcher checked whether the questionnaire was wrongly written or omitted and corrected in time.

### Statistical Analysis

Categorical variables were expressed as numbers, and continuous variables were presented as the mean and standard deviation. Continuous variables were compared using *t*-test. By using univariate and multivariate regression analyses, we compared the differences in scores between RFA group and surgery group. The age, sex, medical expense, and interval time were adjusted in multivariate model. All *p*-values were two-sided, and *p* < 0.05 was considered as statistically significant difference. The SPSS statistical software (version 24.0; IBM, Inc., Chicago, IL) was used to perform all statistical analyses, and the figures were generated using Graph Pad Prism 8.0 (Graph Pad Software, Inc., San Diego, CA).

## Results

### Baseline Characteristics of the Patients

Baseline characteristics had no difference between the RFA group and surgery group in age, sex, BMI, marital status, education level, employment status, comorbidity, family history of thyroid cancer, place to live, and LT4 supplementation. However, in comparison to the patients in the RFA group, patients who underwent surgery were more likely to be patients who can receive medical reimbursement. The time interval from operation to questionnaires completion was also significantly different (5.57 months vs. 20.29 months, *p* < 0.001; Table 2). Meanwhile, there was no significant difference in the baseline characteristics of patients in the unilateral lobectomy group and the total lobectomy group (all *p* > 0.05, Table 2).

**Table 2:** Baseline characteristics of papillary thyroid microcarcinoma patients in RFA group and surgery group.

	**Ablation group (*n* = 54)**	**Surgery group (*n* = 34)**	***p*-value**	**Subgroup of surgery**		***p*-value**
				**Lateral lobectomy (*n* = 18)**	**Total thyroidectomy (*n* = 16)**	
Age(years)	41.89 ± 10.21	42.41 ± 9.86	0.813	42.00 ± 10.30	42.88 ± 9.66	0.801
Sex			0.104			
Male	12	3		2	1	
Female	42	31		16	15	
BMI	23.65 ± 2.51	25.11 ± 3.98	0.061	26.29 ± 3.79	23.79 ± 3.87	0.067
Marital status			0.987			0.900
Married/partner	49	30		16	14	
Living alone	5	4		2	2	
Education level			0.381			0.464
College degree or higher	29	15		9	6	
Others	25	19		9	10	
Employment status			0.932			0.180
Employed	37	23		14	9	
Unemployed	17	11		4	7	
Comorbidity			0.755			0.683
None	50	30		15	15	
Yes	4	4		3	1	
Medical expenses			<0.001*			0.855
Public	19	25		13	12	
Self-paying	35	9		5	4	
Family history of thyroid cancer			0.633			0.932
No	52	32		17	15	
Yes	2	2		1	1	
Place to live			0.989			0.732
Urban	46	29		15	14	
Rural areas	8	5		3	2	
LT4 supplementation			0.427			/
No	3	0		0	0	
Yes	51	34		18	16	
Follow-up duration (months)	5.57 ± 5.46	20.29 ± 17.07	<0.001*	24.44 ± 20.02	15.63 ± 11.98	0.126

* *p < 0.05*.

Among the 34 patients who underwent open surgery, two experienced postoperative hoarse voice. One patient recovered 1 month after surgery and another one was still not recovered at the time of questionnaire completion (3 months after surgery). No complications occurred in the RFA group in this study.

In order to find out the factors related to the HRQoL of PTMC patients, univariate analysis was performed. Both sex and medical expense as categorical variables, as well as age and follow-up duration as continuous variables, are associated with many HRQoL parameters (Table 3). Thus, in order to control the interference of confounding factors, the variables for age, sex, medical expenses, and follow-up time were adjusted during the multivariate analysis.

**Table 3:** Factors related to the quality of life of papillary thyroid microcarcinoma patients.

	**Age**			**Sex**			**Medical expense**			**Follow-up duration**		
	**Coef**	**CI**	***p*-value**	**Coef**	**95% CI**	***p*-value**	**Coef**	**95% CI**	***p*-value**	**Coef**	**95% CI**	***p*-value**
**SF-36**												
**PCS**	−0.245	[−0.472 to−0.018]	0.035*	−6.989	[−12.973 to −1.005]	0.023*	5.139	[0.633 to 9.646]	0.026*	0.002	[−0.172 to 0.175]	0.986
PF	−0.047	[−0.172 to 0.079]	0.463	−3.393	[−6.647 to −0.138]	0.041*	1.023	[−1.476 to 3.521]	0.418	0.001	[−0.093 to 0.094]	0.989
RP	−0.237	[−0.717 to 0.244]	0.330	0.068	[−12.740 to 12.877]	0.992	13.068	[3.852 to 22.285]	0.006*	0.199	[−0.159 to 0.557]	0.272
BP	−0.396	[−0.691 to 0.01]	0.009*	−6.436	[−14.454 to 1.581]	0.114	6.216	[0.244 to 12.187]	0.042*	−0.112	[−0.339 to 0.116]	0.332
GH	−0.293	[−0.679 to 0.093]	0.135	−16.067	[−25.834 to −6.300]	0.002*	0.250	[−7.538 to 8.038]	0.949	−0.062	[−0.353 to 0.229]	0.674
**MCS**	−0.104	[−0.376 to 0.167]	0.448	0.989	[−6.230 to 8.209]	0.786	−1.594	[−7.015 to 3.827]	0.560	0.016	[−0.187 to 0.219]	0.875
VT	0.009	[−0.349 to 0.368]	0.958	−3.507	[−12.975 to 5.961]	0.464	−5.455	[−12.501 to 1.592]	0.128	−0.104	[−0.371 to 0.162]	0.439
SF	−0.117	[−0.380 to 0.147]	0.381	−4.769	[−11.709 to 2.171]	0.176	5.555	[0.415 to 10.695]	0.034*	−0.087	[−0.283 to 0.110]	0.384
RE	−0.136	[−0.734 to 0.462]	0.653	−0.822	[−16.699 to 15.056]	0.918	9.848	[−1.905 to 21.602]	0.099	0.065	[−0.381 to 0.511]	0.773
MH	−0.024	[−0.321 to 0.273]	0.873	−4.026	[−11.846 to 3.795]	0.309	−0.182	[−6.099 to 5.735]	0.951	−0.277	[−0.490 to −0.063]	0.012*
**THYCA-QoL**												
Neuromuscular	0.305	[0.087 to 0.523]	0.007*	−1.481	[−7.510 to 4.547]	0.626	0.000	[−4.540 to 4.54.]	1.000	0.104	[−0.064 to 0.273]	0.222
Voice	0.180	[−0.075 to 0.435]	0.164	−4.718	[−11.481 to 2.045]	0.169	1.515	[−3.618 to 6.648]	0.559	−0.099	[−0.290 to 0.093]	0.309
Concentration	0.154	[−0.094 to 0.402]	0.220	1.065	[−5.562 to 7.692]	0.750	−1.515	[−6.491 to 3.461]	0.547	0.043	[−0.144 to 0.229]	0.651
Sympathetic	0.028	[−0.284 to 0.341]	0.857	−0.944	[−9.223 to 7.335]	0.821	1.515	[−4.705 to 7.734]	0.630	0.052	[−0.181 to 0.284]	0.661
Throat/mouth	−0.008	[−0.247 to 0.232]	0.950	−0.223	[−6.574 to 6.128]	0.944	−1.010	[−5.782 to 3.762]	0.675	0.058	[−0.120 to 0.236]	0.519
Psychological	0.217	[−0.073 to 0.506]	0.141	9.064	[1.530 to 16.599]	0.019*	−3.598	[−9.398 to 2.203]	0.221	0.100	[−0.118 to 0.318]	0.364
Sensory	0.174	[−0.131 to 0.479]	0.259	5.556	[−2.504 to 13.615]	0.174	−7.197	[−13.126 to 1.267]	0.018*	0.048	[−0.181 to 0.277]	0.678
Problems with scar	0.099	[−0.251 to 0.448]	0.576	5.144	[−4.070 to 14.358]	0.270	−2.272	[−9.234 to 4.690]	0.518	0.047	[−0.214 to 0.307]	0.723
Felt chilly	−0.101	[−0.634 to 0.432]	0.707	9.102	[−4.901 to 23.105]	0.200	−2.273	[−12.895 to 8.349]	0.672	0.119	[−0.278 to 0.516]	0.553
Tingling hands/feet	0.175	[−0.077 to 0.426]	0.171	0.578	[−6.162 to 7.319]	0.865	−0.758	[−5.825 to 4.310]	0.767	0.099	[−0.090 to 0.287]	0.300
Gained weight	−0.075	[−0.447 to 0.298]	0.691	−0.487	[−10.372 to 9.398]	0.922	−3.030	[−10.436 to 4.376]	0.418	0.222	[−0.052 to 0.496]	0.112
Headache	−0.044	[−0.414 to 0.326]	0.812	5.205	[−4.538 to 14.949]	0.291	2.273	[−5.087 to 9.632]	0.541	0.005	[−0.271 to 0.281]	0.971
Less interest in sex	−0.436	[−0.921 to 0.049]	0.078	−5.662	[−18.698 to 7.374]	0.390	−12.121	[−21.618 to −2.623]	0.013*	0.220	[−0.145 to 0.585]	0.234
**FoP-Q-SF**												
Physical health	−0.004	[−0.019 to 0.011]	0.621	0.481	[0.096 to 0.866]	0.015*	−0.292	[−0.585 to 0.001]	0.051	−0.004	[−0.015 to 0.007]	0.466
Social family	0.003	[−0.010 to 0.016]	0.696	0.308	[−0.031 to 0.647]	0.074	−0.254	[−0.507 to 0.000]	0.050	0.001	[−0.009 to 0.010]	0.875

* *p < 0.05*.

### SF-36 Questionnaire Scores

**Figure 2:**
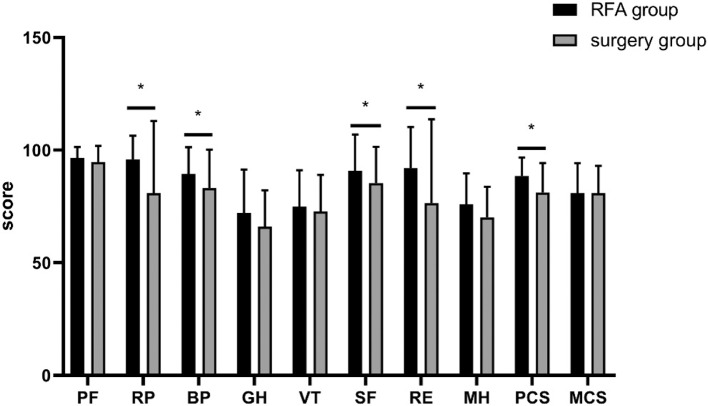
SF-36 score comparison between patients with PTMC in the RFA group and in the surgery group: the patients in the RFA group showed significantly higher scores than the surgery group in 4 domains of HRQoL (*p < 0.05).

The RP, BP, SF, RE, and PCS scores of patients in the RFA group were clearly higher than that in the surgery group (Figure 2). In both univariate and multivariate analyses, the RP, RE, and PCS scores showed a significant negative linear association between RFA group and the surgery group: RP (coefficient [coef] −22.613 [confidence interval (CI) −33.504 to −11.723], *p* < 0.001, RE (coef: −21.901 [CI −36.737 to −7.064], *p* = 0.004), and PCS (coef: −8.312 [CI −13.694 to −2.930], *p* = 0.003). The results suggested that the physical wellbeing of patients in the RFA group was better than in the surgery group (Table 4). In addition, RP, SF, and PCS scores of patients in the unilateral lobectomy group were clearly higher than that in the total thyroidectomy (Table 5).

**Table 4:** Comparison of quality of life in patients with papillary thyroid microcarcinoma who underwent ablation vs. those who underwent surgery.

	**Ablation group**	**Surgery group**	***p*-value**	**Univariate analysis**			**Multivariate analysis**		
				**Coef**	**CI**	***p*-value**	**Coef**	**CI**	***p*-value**
**SF-36 (THE HIGHER SCORE, THE BETTER QUALITY OF LIFE)**									
**PCS**	88.48 ± 8.21	81.18 ± 13.06	0.005*	−7.295	[−11.795 to −2.795]	0.002*	−8.312	[−13.694 to −2.930]	0.003*
PF	96.57 ± 4.84	94.71 ± 7.17	0.186	−1.868	[−4.412 to 0.676]	0.148	−1.981	[−5.194 to 1.232]	0.224
RP	95.83 ± 10.58	80.88 ± 32.01	0.012*	−14.951	[−24.309 to −5.593]	0.002*	−22.613	[−33.504 to −11.723]	<0.001*
BP	89.42 ± 11.87	83.15 ± 17.09	0.045*	−6.270	[−12.407 to −0.132]	0.045*	−3.918	[−11.411 to 3.575]	0.301
GH	72.09 ± 19.24	66.00 ± 16.18	0.128	−6.093	[−13.983 to 1.798]	0.128	−5.524	[−15.087 to 4.038]	0.254
**MCS**	80.92 ± 13.25	80.92 ± 12.08	0.780	−0.787	[−6.363 to 4.788]	0.780	−2.429	[−9.601 to 4.743]	0.502
VT	74.91 ± 15.49	72.79 ± 18.76	0.568	−2.113	[−9.434 to 5.208]	0.568	−2.881	[−12.129 to 6.366]	0.537
SF	90.74 ± 10.29	85.30 ± 14.66	0.044*	−5.446	[−10.736 5 to −0.156]	0.044*	−3.730	[−10.435 to 2.976]	0.272
RE	92.00 ± 18.24	76.47 ± 37.18	0.029*	−15.505	[−27.308 to −3.701]	0.011*	−21.901	[−36.737 to −7.064 ]	0.004*
MH	76.00 ± 13.70	70.12 ± 13.58	0.053	−5.882	[−11.826 to 0.061]	0.053	−2.995	[−10.489 to 4.500]	0.429
**THYCA-QoL (THE LOWER SCORE, THE BETTER QUALITY OF LIFE)**									
Neuromuscular	10.70 ± 11.00	12.42 ± 10.15	0.464	1.719	[−2.929 to 6.366]	0.464	0.786	[−4.937 to 6.509]	0.785
Voice	8.02 ± 12.43	5.88 ± 11.52	0.420	−2.142	[−7.404 to 3.119]	0.420	0.070	[−6.577 to 6.716]	0.983
Concentration	5.86 ± 10.81	7.35 ± 13.10	0.564	1.448	[−3.623 to 6.599]	0.564	0.651	[−5.913 to 7.214]	0.844
Sympathetic	13.58 ± 13.76	16.67 ± 15.89	0.337	3.086	[−3.275 to 9.448]	0.337	4.660	[−3.542 to 12.861]	0.262
Throat/mouth	12.96 ± 11.37	15.36 ± 10.95	0.332	2.396	[−2.482 to 7.274]	0.332	2.235	[−4.089 to 8.558]	0.484
Psychological	15.43 ± 13.68	20.83 ± 13.33	0.072	5.401	[−0.495 to 11.297]	0.072	3.439	[−3.912 to 10.790]	0.355
Sensory	13.89 ± 14.01	18.63 ± 14.66	0.133	4.739	[−1.470 to 10.948]	0.133	2.571	[−5.189 to 10.332]	0.512
Problems with scar	5.56 ± 12.54	13.72 ± 20.30	0.022*	8.170	[1.220 to 15.119]	0.022*	10.246	[1.330 to 19.162]	0.025*
Felt chilly	21.60 ± 22.58	25.49 ± 28.50	0.480	3.885	[−7.002 to 14.772]	0.480	1.205	[−12.774 to 15.184]	0.864
Tingling hands/feet	4.32 ± 11.30	5.88 ± 12.90	0.552	1.561	[−3.635 to 6.757]	0.552	−0.012	[−6.651 to 6.626]	0.997
Gained weight	12.34 ± 16.25	19.61 ± 18.56	0.057	7.262	[−0.212 to 14.735]	0.057	5.803	[−3.842 to 15.449]	0.235
Headache	9.88 ± 15.36	12.74 ± 20.12	0.452	2.869	[−4.680 to 10.418]	0.452	4.543	[−5.131 to 14.217]	0.353
Less interest in sex	17.28 ± 21.22	29.41 ± 24.30	0.016*	12.128	[2.357 to 21.899]	0.016*	10.754	[−1.265 to 22.773]	0.079
**FoP-Q-SF (THE LOWER SCORE, THE BETTER QUALITY OF LIFE)**									
Physical health	1.82 ± 0.68	2.00 ± 0.74	0.285	0.165	[−0.140 to 0.471]	0.285	0.145	[−0.227 to 0.518]	0.440
Social family	1.54 ± 0.59	1.72 ± 0.64	0.185	0.177	[−0.086 to 0.441]	0.185	0.108	[−0.224 to 0.439]	0.520

* *p < 0.05*.

**Table 5:** Comparison of quality of life in patients with papillary thyroid microcarcinoma who underwent unilateral lobectomy vs. those who underwent total thyroidectomy.

	**Lateral lobectomy (*n* = 18)**	**Total thyroidectomy (*n* = 16)**	***p*-value**
**SF-36**			
**PCS**	86.15 ± 8.55	75.59 ± 15.14	0.022*
PF	95.83 ± 5.75	93.44 ± 8.51	0.339
RP	95.83 ± 12.86	64.06 ± 38.70	0.006*
BP	84.72 ± 18.89	81.38 ± 15.22	0.577
GH	68.22 ± 15.05	63.50 ± 17.52	0.404
**MCS**	79.68 ± 14.27	80.63 ± 9.46	0.822
VT	78.33 ± 17.06	66.56 ± 19.12	0.067
SF	90.12 ± 12.57	79.86 ± 15.30	0.040*
RE	87.04 ± 23.26	64.59 ± 46.30	0.094
MH	71.33 ± 14.34	68.75 ± 13.00	0.588
**THYCA-QoL**			
Neuromuscular	10.49 ± 11.09	14.58 ± 8.81	0.247
Voice	9.26 ± 14.26	2.08 ± 5.69	0.062
Concentration	4.63 ± 9.58	10.42 ± 15.96	0.219
Sympathetic	12.96 ± 12.20	20.83 ± 18.76	0.165
Throat/mouth	16.67 ± 12.78	13.89 ± 8.61	0.469
Psychological	14.81 ± 10.13	27.60 ± 13.51	0.004*
Sensory	16.67 ± 12.8	20.83 ± 16.67	0.416
Problems with scar	9.26 ± 19.15	18.75 ± 20.97	0.177
Felt chilly	25.93 ± 35.34	25.00 ± 19.24	0.924
Tingling hands/feet	3.70 ± 10.78	8.33 ± 14.91	0.314
Gained weight	16.67 ± 17.15	22.92 ± 20.07	0.335
Headache	11.11 ± 19.80	14.58 ± 20.97	0.623
Less interest in sex	31.48 ± 24.18	27.08 ± 25.00	0.606
**FoP-Q-SF**			
Physical health	1.86 ± 0.75	2.14 ± 0.72	0.287
Social family	1.60 ± 0.60	1.86 ± 0.68	0.255

* *p < 0.05*.

### THYCA-QOL Questionnaire Scores

**Figure 3:**
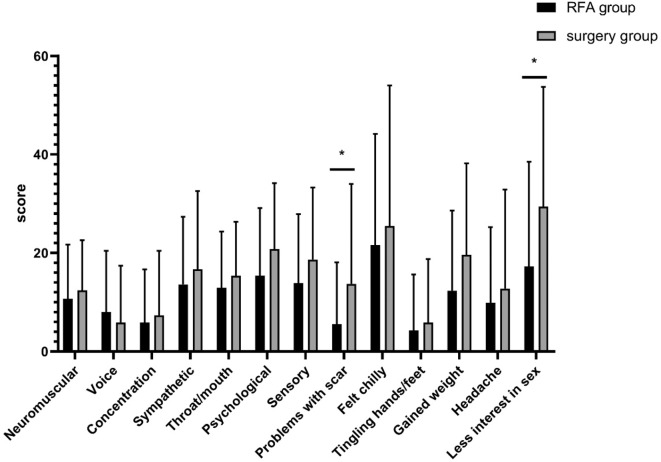
THYCA-QoL score comparison between patients with PTMC in the RFA group and in the surgery group: the patients in the RFA group showed significantly lower scores than the surgery group in 2 domains of HRQoL (*p <0.05).

The “problems with scarring” and “less interest in sex” scale scores of patients in the RFA group were lower than in the surgery group, indicating a lower level of complaint relating to symptom in the RFA group (Table 4, Figure 3). In both univariate and multivariate analyses, the “problems with scarring” scale score showed a significant positive linear association between groups (coef: 10.246 [CI 1.330 to 19.162], *p* = 0.025 according to the multivariate analysis). The “less interest in sex” scale score showed a significant difference between the two groups in the univariate analysis but in the multivariate analysis there was no significant difference (Table 4). Psychological scores were clearly higher in the unilateral lobectomy group than in the total thyroidectomy (Table 5).

### FoP-Q-SF Questionnaire Scores

Neither physical health nor social family domain had significant differences in FoP-Q-SF questionnaire scores between the two groups in all analyses (*p* > 0.05) (Table 4).

## Discussion

With the development of medicine, the definition of health has been changing (41), which is defined as a combination of physical, emotional, and social potential, not simply the absence of disease. Additionally, HRQoL reflects personal satisfaction or happiness with their life, and to some extent it may affect or be affected by a certain aspect of the “health” definition mentioned above. Thus, HRQoL has been introduced as an assessment of an individual's health status. The recent ATA Guidelines also emphasize the importance of long-term HRQoL outcomes when physicians make treatment decisions (2).

Our study evaluated the HRQoL of patients with PTMC under different treatment strategies. Several significant differences were found in parameters of the SF-36 questionnaires between the RFA group and the surgery group. After adjusting for age, sex, medical expense, and follow-up duration, the surgery group reported more problems associated with PCS, RP, and RE than the RFA group. RP and RE represent restrictions on daily activities or work due to physical and emotional effects, respectively. PCS represents the overall physical health of the patients. Therefore, patients in the surgery group suffered more adverse effects than those in the RFA group, especially for those who underwent total thyroidectomy. The result is consistent with the results of Lubitz CC et al., which reported that the domain of RE remained decreased years after surgery even without the recurrence of PTC (42).The cause may be due to surgical trauma or complications (43). In general, the greater the surgical trauma, the more likely it is to cause complications (44, 45). According to research reports, the probability of permanent recurrent laryngeal nerve injury after surgery is 0 to 12%, and the probability of permanent hypocalcemia is 0 to 7% (46–48). However, RFA seems to present fewer complications (48). In our study, no patients in the RFA group reported complications. Therefore, we believe that the smaller the trauma, the lower the possibility of complications and the higher the quality of life in patients with PTMC.

In addition, the THYCA-QoL questionnaire used in our study had an item about scarring problems. The problem of scarring was more common in patients who underwent surgery than those who underwent RFA. This was one of the major causes of quality of life decline in the surgery group. Previous studies had revealed that an obvious scar may negatively affect the HRQoL of PTC patients (49) because of the majority of women with thyroid cancer and a good prognosis, a significant number of patients are concerned about permanent and unsightly scars. Although surgery was recommended, the concerns about scarring may affect the HRQoL of survivors from seemly minor problems such as difficulties in choosing clothes to more significant problem such as avoiding communicating with others and developing an inferiority complex, even influencing their career development (50). Additionally, quite a lot of patients may think that the apparent scar may have caused damage to their body image, which is a definition of an individual's subjective view of their own body and has to do with self-esteem and self-perception, closely related with HRQoL.

Some studies had revealed that patients with post-treatment thyroid cancer are constantly concerned about recurrence and metastasis during long-term follow up (51). Hedman et al. reported that only 7% of patients actually experience disease recurrence, but up to 48% of thyroid cancer patients are under pressure to worry about recurrence, which has seriously affected their quality of life (52). The perceptions of the disease from thyroid cancer survivors are often subjective and emotional, and may be inconsistent with the actual severity of the disease. However, in our study, it had no significant difference between the RFA group and the surgery group in the analysis of anxieties and fears associated with disease progression using the FoP-Q-SF questionnaire. This reveals that the patients in the RFA group were not more concerned about the progression of the disease although ablative therapy was only localized to the lesion, which was one of the main concerns during the follow-up of patients who underwent RFA. However, the patients in the RFA group reported less interest in sexual activity with no statistical significance after adjustment in the multivariate analysis, which may be associated with some anxiety resulting from cancer itself (53).

The three questionnaires used in this study have been demonstrated by previous studies to be validated in evaluating patients' HRQoL. Among them, SF-36 is considered to be a commonly used and sensitive instrument in measuring HRQoL in thyroid cancer in previous studies (54). Gou J et al. reported that RP, RE, and PCS of SF-36 were the factors associated with the HRQoL of patients with PTMC (43). These results are consistent with our study. It indicates that SF-36 is an appropriate tool to evaluate HRQoL of thyroid cancer survivors. However, the SF-36 cannot evaluate all aspects of HRQoL such as disease symptoms or treatment side effects. Thus, the THYCA-QoL questionnaire and FoP-Q-SF were included as a reasonable complement to evaluate important aspects regarding thyroid cancer-specific symptoms (7) and fear of disease progression, which may be the strong determinants of the quality of life after thyroid cancer.

Since the strategy of RFA was first introduced in patients with PTMCs, many studies reported its safety and efficacy for treating low-risk PTMCs (26, 55). Our study suggests that RFA may have advantages in improving the HRQoL of patients with no relationship to anxiety or fear associated with disease progression.

This study has several limitations. First, the baseline characteristics of patients between the two treatment groups were not all matched even though the multivariate analysis was adjusted for patients' follow-up duration, which was a key factor in the assessment of HRQoL (56) as well as medical expense (57). Thus, the results of this study can be biased. Second, the number of patients included in our study was limited. Third, the follow-up time is not long enough, which may overestimate the negative impact of surgery to HRQoL (58), since the HRQoL of cancer patients may improve over time after surgery (7). Last, preoperative quality of life was unknown in both groups. Thus, prospective studies with large samples and longer-term follow-up are proposed.

In conclusion, our study suggested that US-guided RFA offers advantage in terms of HRQoL and supports the role of ablation as an alternative strategy for patients with PTMC except for surgery.

## Data Availability Statement

The data used to support the findings of this study are available from the corresponding author upon request.

## Ethics Statement

The studies involving human participants were reviewed and approved by institutional review board of General Hospital of Chinese PLA (S2019-211-01). The patients/participants provided their written informed consent to participate in this study. Written informed consent was obtained from the individual(s) for the publication of any potentially identifiable images or data included in this article.

## Author Contributions

YLu: integrity of the whole study, analysis of data, and review of final manuscript. YLa and MZ: management of data and manuscript writing. LY and YZ: statistical analysis of data. ZJ and JX: literature review and input of scores in questionnaires. YLa: distribution and recovery of questionnaires, collection and analysis of data.

## Conflict of Interest

The authors declare that the research was conducted in the absence of any commercial or financial relationships that could be construed as a potential conflict of interest.
